# Genomic heterogeneity differentiates clinical and environmental subgroups of *Legionella pneumophila* sequence type 1

**DOI:** 10.1371/journal.pone.0206110

**Published:** 2018-10-18

**Authors:** Jeffrey W. Mercante, Jason A. Caravas, Maliha K. Ishaq, Natalia A. Kozak-Muiznieks, Brian H. Raphael, Jonas M. Winchell

**Affiliations:** Pneumonia Response and Surveillance Laboratory, Respiratory Diseases Branch, Centers for Disease Control and Prevention, Atlanta, GA, United States of America; University of Mississippi Medical Center, UNITED STATES

## Abstract

*Legionella* spp. are the cause of a severe bacterial pneumonia known as Legionnaires’ disease (LD). In some cases, current genetic subtyping methods cannot resolve LD outbreaks caused by common, potentially endemic *L*. *pneumophila* (Lp) sequence types (ST), which complicates laboratory investigations and environmental source attribution. In the United States (US), ST1 is the most prevalent clinical and environmental Lp sequence type. In order to characterize the ST1 population, we sequenced 289 outbreak and non-outbreak associated clinical and environmental ST1 and ST1-variant Lp strains from the US and, together with international isolate sequences, explored their genetic and geographic diversity. The ST1 population was highly conserved at the nucleotide level; 98% of core nucleotide positions were invariant and environmental isolates unassociated with human disease (n = 99) contained ~65% more nucleotide diversity compared to clinical-sporadic (n = 139) or outbreak-associated (n = 28) ST1 subgroups. The accessory pangenome of environmental isolates was also ~30–60% larger than other subgroups and was enriched for transposition and conjugative transfer-associated elements. Up to ~10% of US ST1 genetic variation could be explained by geographic origin, but considerable genetic conservation existed among strains isolated from geographically distant states and from different decades. These findings provide new insight into the ST1 population structure and establish a foundation for interpreting genetic relationships among ST1 strains; these data may also inform future analyses for improved outbreak investigations.

## Introduction

*Legionella* is a globally important cause of severe and sometimes fatal bacterial pneumonia known as Legionnaires’ disease (LD). Approximately 80% of laboratory diagnosed LD in the United States (US) is due to a single species, *L*. *pneumophila* (Lp), and up to 79% of Lp infections are attributable to serogroup 1 (Lp1) [1, 2]. Thus, molecular comparison of clinical and environmental isolates is helpful for source attribution during LD outbreaks; at least two laboratory techniques, pulsed-field gel electrophoresis (PFGE) and sequence-based typing (SBT), have been widely used for this purpose. While both are currently in use, SBT became the gold standard for *L*. *pneumophila* DNA molecular typing over the past decade, allowing for universal exchange of sequence type (ST) information.

Nevertheless, SBT is unable to differentiate Lp strains with locally prevalent STs, thus creating uncertainty around the interpretation of isolate genetic relationships. In the US, the single largest category of Lp1 strains isolated from cases of sporadic disease between 1982 and 2012 and sent to the Centers for Disease Control and Prevention (CDC) was ST1 (25%) [3]. The same report found that 49% of environmental Lp1 isolated from US facilities with no known disease association were ST1. The clinical and environmental presence of ST1 is not unique to the US but has been reported widely, including in Canada [4–6], England and Wales [7], mainland Europe [8–16], the Middle East [17, 18], Australia [19], and several countries in Asia [20–24]. It has been proposed that ST1 “Paris” group strains (i.e., isolates with Lp strain Paris-like PFGE and microarray patterns) may represent a homogeneous Lp subpopulation [25], thus requiring high-resolution genetic analyses to uncover rare polymorphisms among isolates. Several recent publications have reported analyses of recombination and background mutation among non-clonal ST1 datasets and between other frequently encountered sequence types [25–28]. Yet, very little is known of the ST1 population genetic structure and ecological diversity in either clinical or man-made environmental settings.

As demonstrated in several recent publications [19, 27, 29–31], whole-genome sequencing (WGS) can deliver resolving power beyond traditional typing methods for high-confidence discrimination of outbreak-associated isolates. Thus far, no study has characterized a large, geographically diverse ST1 isolate collection composed of clinical and environmental subgroups to define its population structure. In the present study, we genetically compared a large collection of clinical and environmentally-derived ST1 strains in the CDC archive and from international locations. We also investigated how nucleotide variability was distributed throughout the US ST1 population and its potential significance.

## Results

### ST1 is prevalent in both US and European *L*. *pneumophila* sequence type collections

**Fig 1:**
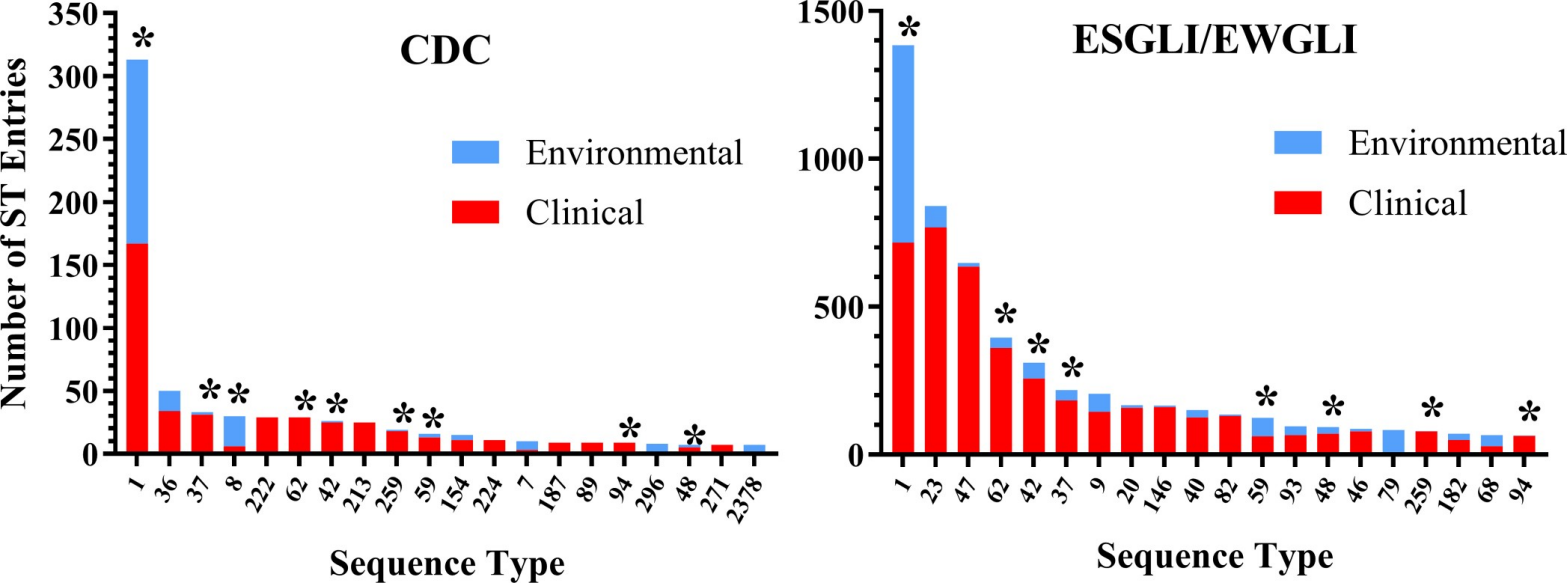
The top 20 most prevalent environmental and clinical-associated *L*. *pneumophila* sequence types in the ESGLI and U.S. CDC SBT databases. Data current as of December 2017. Stars highlight sequence types common to both top 20 collections.

As of December, 2017, 250 unique sequence types were identified among 1,033 sporadic clinical and non-outbreak-associated environmental isolates subjected to SBT from the CDC *Legionella* Laboratory *L*. *pneumophila* isolate collection. The top 20 most prevalent STs in the European Study Group for *Legionella* Infections (ESGLI) SBT database (http://www.hpa-bioinformatics.org.uk/legionella/legionella_sbt/php/sbt_homepage.php) and CDC collections (Fig 1) represent 49% and 65% of all deposited strains, respectively. Among all STs not associated with outbreaks, the largest single subpopulation is composed of ST1 isolates in both collections (ESGLI = 13%, n = 1,391; US = 31%, n = 315). By source, ST1 strains are found in higher proportions of both clinical and environmental isolates in the US (23%, n = 167 and 51%, n = 146 respectively) compared to the ESGLI database (10%, n = 717 and 18%, n = 667), however, environmental isolates are likely subject to greater sampling bias. Nonetheless, ST1 is the most common environmental sequence type in both the ESGLI and CDC collections, the most common sporadic clinical ST in the US, and the second most prevalent clinical ST in the ESGLI database, just ahead of ST47 (9%), but behind ST23 (11%).

The frequency of ST1 recovery from clinical and environmental sources in the US and Europe contrasts sharply with the small number of LD outbreaks attributed to this ST, as previously reported [3]. In the US, only 4 out of 38 LD outbreaks investigated by the CDC have been linked to ST1 since 1982. While not confirmed, this relationship appears to hold true in the Europe as well, where a large proportion of reported LD outbreaks are due to sequence types other than ST1 [32–39]. Further analysis of the ESGLI and CDC ST1 clonal complexes and single and double locus variants are provided in the S1 Results.

### Distinct ST1 subgroup pangenomes contain unique genetic content

**Fig 2:**
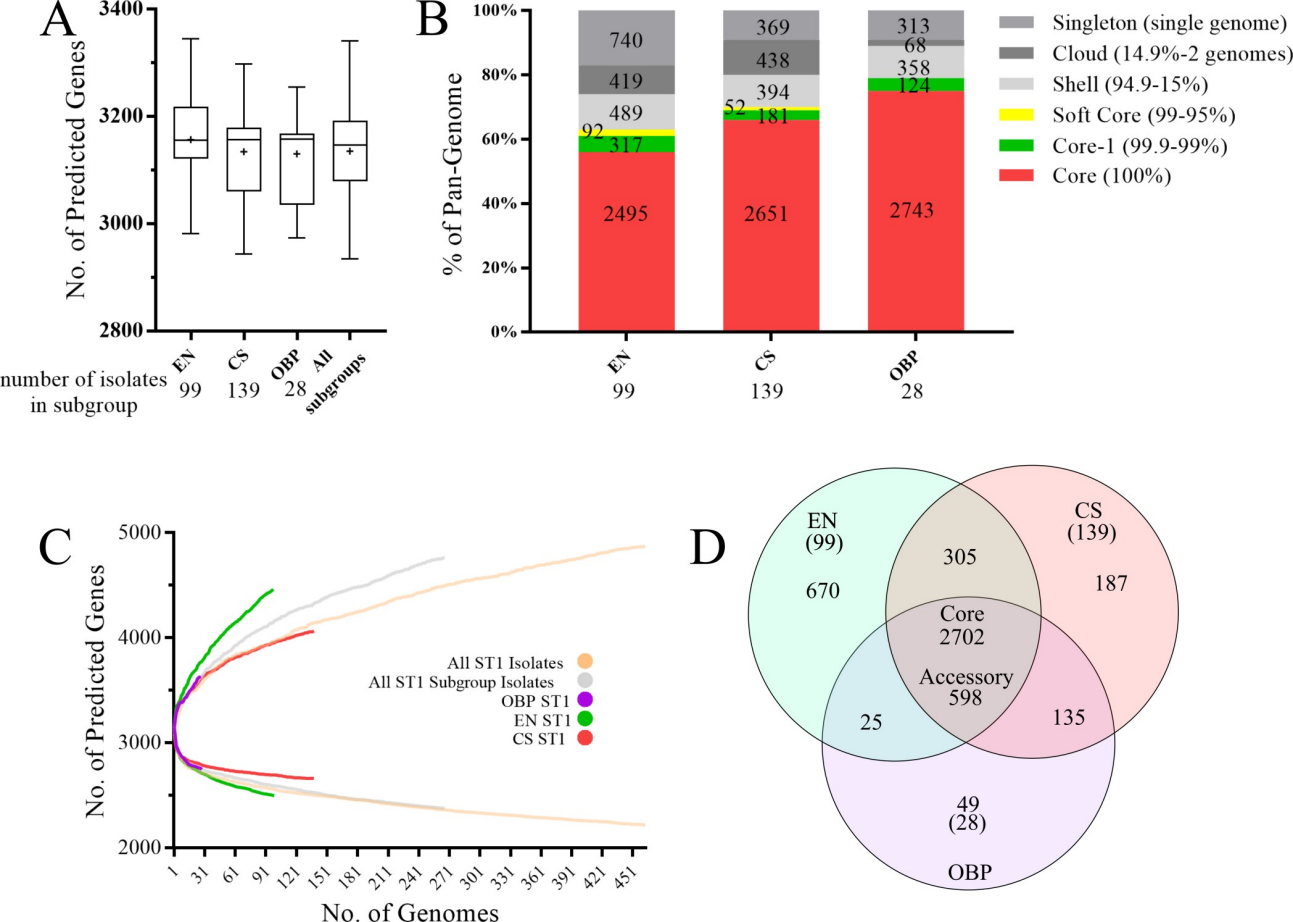
*L*. *pneumophila* ST1 isolate core and pangenome description and subgroup comparisons. **A)** Average number of predicted genes among EN, CS, and OBP subgroups. Box-and-whisker plots display the minimum, maximum, 25^th^/75^th^ percentiles, median (horizontal line), and mean (plus sign) **B)** Predicted pangenome comparison. Gene frequency categories (to the right of the graph) are based, in part, on the ‘roary_plots’ output visualization of Roary, with the addition of a ‘Core-1’ category. Numbers within each stacked bar plot represent the gene count for each frequency category. **C)** Rarefaction curves for pan and conserved genomes. **D)** Direct comparison of predicted pangenomes showing the size of intersecting and unique accessory (non-core or core-1) genomes. The number of isolate genomes included in each subgroup is displayed either at the bottom of the subgraph (A and B) or in parentheses below the subgroup abbreviation (D). Abbreviations: ‘CS’, clinical sporadic; ‘EN’, environmental; ‘OB/OBP’, outbreak and potential outbreak associated.

For the purpose of genetic comparison, ST1 isolate genomes in the current study were categorized into environmental (EN) which are unassociated with known cases of disease, clinical sporadic (CS), and either outbreak (OB) or outbreak/potential outbreak-associated (OBP) subgroups based on their source of origin. Among all ST1 subgroups, the median number of predicted genes (3156–3158 genes) did not vary appreciably (Fig 2A), however, the average number of genes per isolate was highest in the EN subgroup compared to the CS and OBP subgroups (EN = 3,157; CS = 3,135; and OBP = 3,131 genes), which is consistent with the larger average EN genome size (EN = 3,614,970, CS = 3,577,094, and OBP = 3,564,149 bp). While a small OBP pangenome of 3,606 genes was expected (because this subgroup included only 28 genomes; Fig 2B), it is remarkable that the EN pangenome (4,606 genes) was discernibly larger (up to 24%) than the CS pangenome (4,033 genes) despite the EN subgroup including fewer isolates than the CS subgroup (EN = 99 genomes, CS = 139 genomes). The inclusion of unequal numbers of genomes in each subgroup could skew pangenome observations, however, the EN subgroup displayed the steepest positive slope on a pangenome rarefaction curve (Fig 2C), indicating that accessory genes continue to accumulate. The CS pangenome curve, in contrast, has begun to plateau, suggesting a closed pangenome and that EN/CS pangenome differences stated above may be *underestimations*. The OBP pangenome rarefaction curve tracked with the CS or combined ST1 subgroup, but this curve should be interpreted with caution due to the limited number of isolate sequences. The EN accessory genome (1,740 genes), which includes all gene frequency categories except ‘core’ and ‘core-1’, was also dramatically larger than that of the CS (1,253 genes) and OBP (739 genes) subgroups. A large proportion of genes in the EN (43%), CS (30%), and OBP (42%) accessory genomes were found in only one isolate of the subgroup (‘singletons’), and up to ~33% of these may be orthologs or paralogs of existing genes in the dataset.

The underlying genetics of the ST1 subgroups were investigated through gene ontology (GO) classification; significant term enrichment (p<0.05) was noted within and among some individual pangenomes and subgroups (S1 Results, S2 Fig, and S2 Table). All ST1 subgroup pangenomes shared a common 598 gene accessory repertoire (genes found in at least 1 isolate of all subgroups; S3 Table and Fig 2D) that was enriched for terms encompassing conjugal DNA transfer and transposition-related factors. The EN-specific accessory genome (670 genes), the largest of the 3 subgroups, was enriched for GO terms related to transposition and recombination (e.g., XerCD recombinases), restriction-modification (e.g., Type I restriction enzymes), gene regulation (e.g., *csrA* and *lexA*) and toxin-antitoxin systems (e.g., DinJ and ParD1). The CS-specific accessory genome (187 genes) was enriched for only a single term housing genes devoted to signal transduction (e.g., *fixJ*/*fixL*, and the *yegE* diguanylate cyclase).

Gene-level subgroup analysis further revealed fifteen predicted genes that were enriched in the EN subgroup (p<0.05; Table 1). Most genes (13 of 15) were included in two groupings distributed across the CS and EN subpopulations, and assembled in close physical proximity on the same contig, suggesting linkage. No predicted genes in the CS subgroup met the threshold for enrichment. However, among the top 10 genes with the lowest p values (p = 0.086–0.2), half appeared to be physically linked (Table 1, Group 3) and were either annotated or homologous to known acetyltransferases, including the potential virulence associated *lag-1* gene [40], which was detected in ~37% of CS isolates (and in ~19% and ~54% of EN and OBP isolates, respectively). Additional details of gene groups, and GO term and gene level enrichment analyses are provided in the Supporting Results (S1 Results).

**Table 1:** Genes enriched or near enrichment in ST1 subgroups.

Enriched or Depleted Genes in the EN and CS Subgroups			CS Isolates			EN Isolates			
Predicted Gene or Group ID	Annotation/Notes	Group	With ORF	Without ORF	Enrichment p value	With ORF	Without ORF	Enrichment p value	2-tailed Fisher's Exact Test*
*trbC*	conjugal transfer protein TrbC	1	10	128	1	25	70	3.72E-05	0.0401
*trbD*	conjugal transfer protein TrbD	1	10	128	1	25	70	4.71E-05	0.0401
*trbF*	conjugal transfer protein TrbF	1	10	128	1	25	70	7.27E-05	0.0401
*trbJ*	conjugal transfer protein TrbJ	1	10	128	1	25	70	7.27E-05	0.0401
*trbG*	conjugal transfer protein TrbG	1	10	128	1	25	70	7.27E-05	0.0401
*trbL*	conjugal transfer protein TrbL	1	10	128	1	25	70	7.27E-05	0.0401
*trbI/ptlG*	Pertussis toxin liberation protein G/conjugal transfer protein TrbI	1	10	128	1	25	70	7.27E-05	0.0401
*trbB/ptlH*	Pertussis toxin liberation protein H/ATPase TrbB	1	10	128	1	25	70	7.27E-05	0.0401
*traG*	Conjugal transfer protein TraG	1	10	128	1	25	70	7.27E-05	0.0401
*traJ*	Relaxosome protein TraJ	1	10	128	1	25	70	7.27E-05	0.0401
*trbE/virB4*	Type IV secretion system protein *virB4*/conjugal transfer protein TrbE	1	10	128	1	25	70	7.27E-05	0.0401
*xerC*	tyrosine recombinase, XerC	2	4	134	1	17	78	7.27E-05	0.0401
*hin*	DNA-invertase hin/transposon resolvase	2	4	134	1	18	77	7.27E-05	0.0401
group_2725	hypothetical protein		1	137	1	12	83	0.000106	0.0401
group_18	Transposase		75	63	1	76	19	0.000116	0.0401
**Top 10 Most Enriched Genes in CS Subgroup**									
group_2156	lpp2968, hypothetical protein	3	135	3	0.0004	81	14	1	0.0861
group_2157	lpp2967 hypothetical protein, potential acetyl-CoA-acetyltransferase	3	135	3	0.0008	82	13	0.9999	0.1096
group_1313	lpp2986, putative acetyltransferase	3	135	3	0.0015	83	12	0.9997	0.1096
group_1315	lpp2983, weakly similar to acetyltransferase	3	135	3	0.0017	83	12	0.9998	0.1096
group_2152	lpp2987, hypothetical protein	3	135	3	0.0017	83	12	0.9998	0.1096
group_2153	lpp2986, putative acetyltransferase	3	135	3	0.0017	83	12	0.9998	0.1096
group_2154	lpp2984, hypothetical protein	3	135	3	0.0017	83	12	0.9998	0.1096
group_2136	lpp1951 hypothetical protein		132	6	0.0017	79	16	0.9998	0.1096
*lag*-1	O-acetyltransferase		50	88	0.0030	18	77	0.9989	0.1824
group_1130	lpp0331, putative GIY-YIG nuclease superfamily protein		112	26	0.0031	61	34	0.9988	0.1980

CS = Clinical Sporadic Isolates

EN = Environmental Isolates Unassociated with Known LD

BH = Benjamani-Hochberg Correction Applied

*, Combined EN and CS gene frequencies, BH Corrected

Within the context of gene enrichment, two recent studies [41, 42] identified an efflux pump (LpeAB) found primarily in ST1 strains that confers increased macrolide resistance in *Legionella*. We detected the genes encoding this pump in 501 ST1 and ST1-like isolate sequences in the current dataset and no enrichment was found for one or more particular ST1 subgroup. All but 8 of these isolates shared the same *lpeAB* allele, and a single ST1 SLV from China (‘SZ2012007’, ST752; [26]) did not encode either pump component.

### ST1 genomes are highly conserved but contain discrete regions of nucleotide diversity

The current analyses revealed considerable gene content differences among the EN, CS, and OBP subgroups. Therefore, we investigated nucleotide variation among shared genomic loci by mapping all ST1 isolate sequences to the *L*. *pneumophila* str. Paris (ST1) reference genome. An initial, high-level comparison indicated that the ST1 population was highly conserved; ~41% (1,427,306 nt) of all mapped nucleotide positions were core (conserved in all isolates without missing data or gaps), and ~98% of these core sites were invariant. Of the remaining non-core nucleotide positions, 97.5% were completely conserved across all sequences in which they were included. An average nucleotide diversity (d) of 7.09E-4 (SE±5.60E-6) SNPs/nucleotide was also noted, and consistent with its larger, open pangenome, the EN subgroup contained 62% and 7% greater nucleotide diversity on average (d = 8.78E-4 SE±6.48E-6) compared to the CS (d = 5.46E-4 SE±4.98E-6) and OBP subgroups (d = 8.15E-4 SE±6.45E-6), respectively.

**Fig 3:**
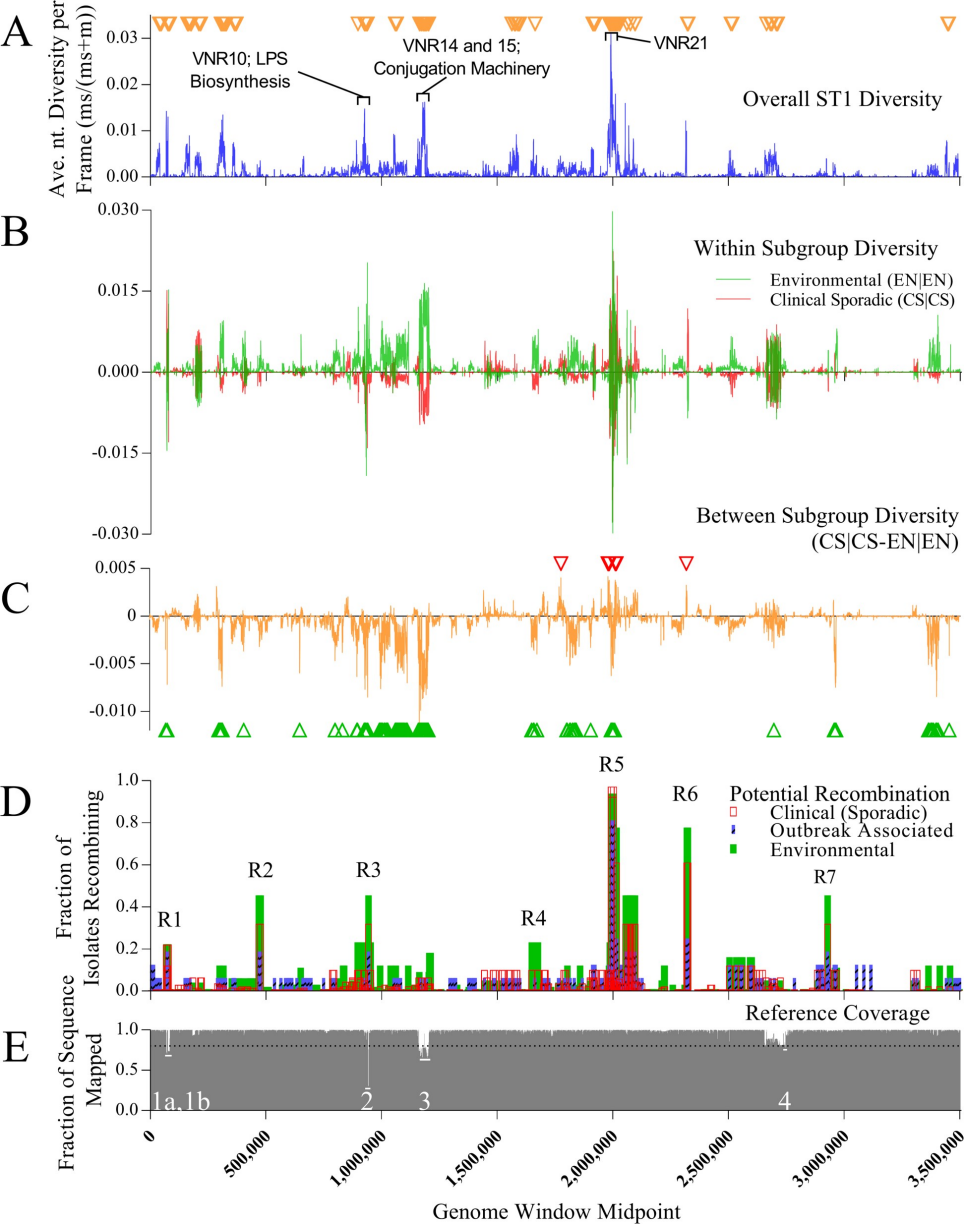
Genetic diversity and potential recombination among ST1 and ST1-like isolate sequences. **A)** Nucleotide diversity of the combined 501 isolate dataset at 500 bp overlapping windows relative to *L*. *pneumophila* str. Paris. Gold inverted triangles indicate windows that meet or exceed the 3% diversity threshold. **B)** Within group nucleotide diversity of the EN and CS subgroups, where the subgroup with the highest diversity at any single window is displayed in the positive Y axis while the subgroup with lower diversity is displayed in the negative Y axis. **C)** Nucleotide diversity between the CS and EN subgroups. Within group CS diversity is subtracted from EN diversity (CS|CS-EN|EN), thus, genomic windows with higher within group CS diversity will be in the positive Y axis while windows with higher EN within group diversity will be in the negative Y axis. Red and green triangles indicate the subgroup (CS or EN, respectively) with comparatively higher nucleotide diversity at that window exceeding the 3% threshold. **D)** Potential recombination frequency for each ST1 subgroup across the genome. Windows with recombination frequencies >20% in any single subgroup are labeled and numbered. **E)** Sequence mapping coverage at each genomic window for all ST1 isolate sequences. Windows or regions with coverage below 80% are labeled and numbered 1–4.

A survey of nucleotide variation across the resulting ST1 alignment (Fig 3A) found that while overall diversity remained low, 33 distinct variable nucleotide regions (VNR) exceeded the top 3% of all diversity measurements, encompassing 190,500 total bases (S4 Table). This threshold was chosen to maximize region contiguity while minimizing the total number of regions. VNRs were an average length of 5,772 nt and contained 183 predicted genetic features including the pP36 mobile element [43] (VNR5 and 6), multidrug transporters (resistance-nodulation-cell division [RND] superfamily efflux pumps; VNR7), and the *L*. *pneumophila* sg1 15kb and 18kb LPS biosynthesis loci (VNR10; Fig 3A “LPS Biosynthesis”) [44, 45], among others. A further description of notable genetic features within the VNRs is contained in the Supporting Results (S1 Results).

Nucleotide variation was generally localized at the same genomic loci in both the EN and CS subgroups, but the magnitude of that variation was typically larger within the EN subgroup (Fig 3B). The EN subgroup contained greater variability in ~50% of all nucleotide windows, compared to ~25% in the CS subgroup. Within-group comparative analysis (EN-CS) identified 34 EN-specific regions encompassing 206,250 bases that met the 3% diversity threshold, but only revealed 4 discrete, high diversity CS regions incorporating 12,750 bases under the same conditions (Fig 3C and S5 Table). Six GO gene categories were enriched within the combined EN variable region set (S6 Table), and a majority of genes in most categories clustered in a single region (EN-VNR13). Further details of EN-specific enrichment is found in the Supporting Results (S1 Results).

### Recombination contributes to genetic variation among ST1 subgroups

To further study the basis of regional nucleotide variation, we searched for areas of increased SNP density across the ST1 multiple sequence alignment (Fig 3D) that could be indicative of recombination. Over 400 potential recombination events were detected, many of which overlapped and were shared between subgroups. More recombination events were identified in the EN (n = 310 events) versus the CS (n = 250) or OB (n = 100) subgroups. The average percentage of isolate sequences within each subgroup recombining across all events was similar between the CS and EN subgroups (4.5% and 4.9%, respectively), but was approximately two-fold higher in the OB category (9.5%). However, the EN subgroup exhibited consistently higher recombination frequencies in a larger percentage (55%) of the 126 exact events shared with the CS subgroup population (38%).

Seven individual or clustered recombination regions (R1-R7 in Fig 3D and Table 2) demonstrated recombination frequencies among isolates above 20%, a threshold chosen to overcome average recombination levels by at least 2 fold in all subgroups. Many of these prominent “hotspots” (e.g., R2, R3c, R5b-R7) exhibited subgroup recombination frequencies ≥ 45%, and their genome coordinates often coincided with one or more VNRs identified in the EN, CS, and/or full ST1 datasets (e.g., R1, R3-R6). For example, R3b and R3c overlapped large portions of the 15kb and 18kb LPS biosynthesis regions, which was consistent with a previous report of nucleotide variability at this locus among sg1 strains [45]. This suggested that recombination, in part, could explain the differences in nucleotide diversity observed among subgroups. Several putative ST1 recombination regions identified here, such as R5b/c which overlaps VNR21, have been at least partially reported in various *L*. *pneumophila* STs, including ST1, confirming their importance as sites of high recombination potential and variability [46]. Two hotspots (R5b/c and R6) exhibited extremely high isolate recombination frequencies (>77.8%) from at least one subgroup. Region R5b/c encoded factors associated with outer membrane protein assembly (e.g, lpp1769, BamA) and porphorin-containing compound biosynthesis (e.g., lpp1771, HemB), among others. Additional descriptions of the R5b/c and R6 regions are provided in the Supporting Results (S1 Results).

**Table 2:** Recombination hotspots in ST1 subgroups with frequencies above 20%.

		Percentage of Isolates in Category with Putative Recombination Event			
Region ID	Coordinates relative to *L*. *pneumophila* str. Paris (bp)	CS	EN	OB	Predicted Features (based on Lp1 str. Paris reference)
R1	68053–76221	22.0%	22.2%	18.8%	lpp0064-lpp0075
R2	467796–476192	31.9%	45.5%	18.8%	lpp0418-lpp0427
R3a	895759–899423	9.9%	23.2%	0.0%	lpp0801-beginning of lpp0802
R3b	923274–951936	9.9%	23.2%	0.0%	lpp0825-lpp0849
R3c	942909–945826	31.9%	45.5%	18.8%	lpp0841-lpp0843
R4	1648686–1675289	9.9%	23.2%	0.0%	lpp1476-lpp1501
R5a	1986115–2001091	9.9%	23.2%	0.0%	lpp1765-lpp1774
R5b	1992558–2003075	97.2%	93.9%	81.3%	lpp1769-lpp1775
R5c	2014953–2018845	61.0%	77.8%	25.0%	lpp1784-lpp1787
R5d	2055666–2064175	31.9%	45.5%	18.8%	lpp1822-lpp1825
R5e	2072723–2076994	31.9%	45.5%	18.8%	lpp1832-lpp1836
R5f	2084600–2094628	31.9%	45.5%	18.8%	lpp1843-lpp1849
R6	2319107–2324375	61.0%	77.8%	25.0%	lpp2053-lpp2058
R7	2928006–2929589	31.9%	45.5%	18.8%	lpp2574-lpp2575

We also determined that, on average, ~98.5% of the *L*. *pneumophila* str. Paris reference genome was covered or mapped by ≥ 80% of ST1 isolate sequences (Fig 3E). However, sequence coverage dipped below 80% at 4 discrete loci, totaling ~55,500 bp, designated low coverage locus 1 through 4 (LC1-4).Thus, sequence mapping coverage may contribute to some low level variability observed within or between subgroups. Additional details of these low coverage regions are provided in the Supporting Results (S1 Results).

### ST1 isolates cluster tightly and demonstrate unexpected phylogenetic relationships

**Fig 4:**
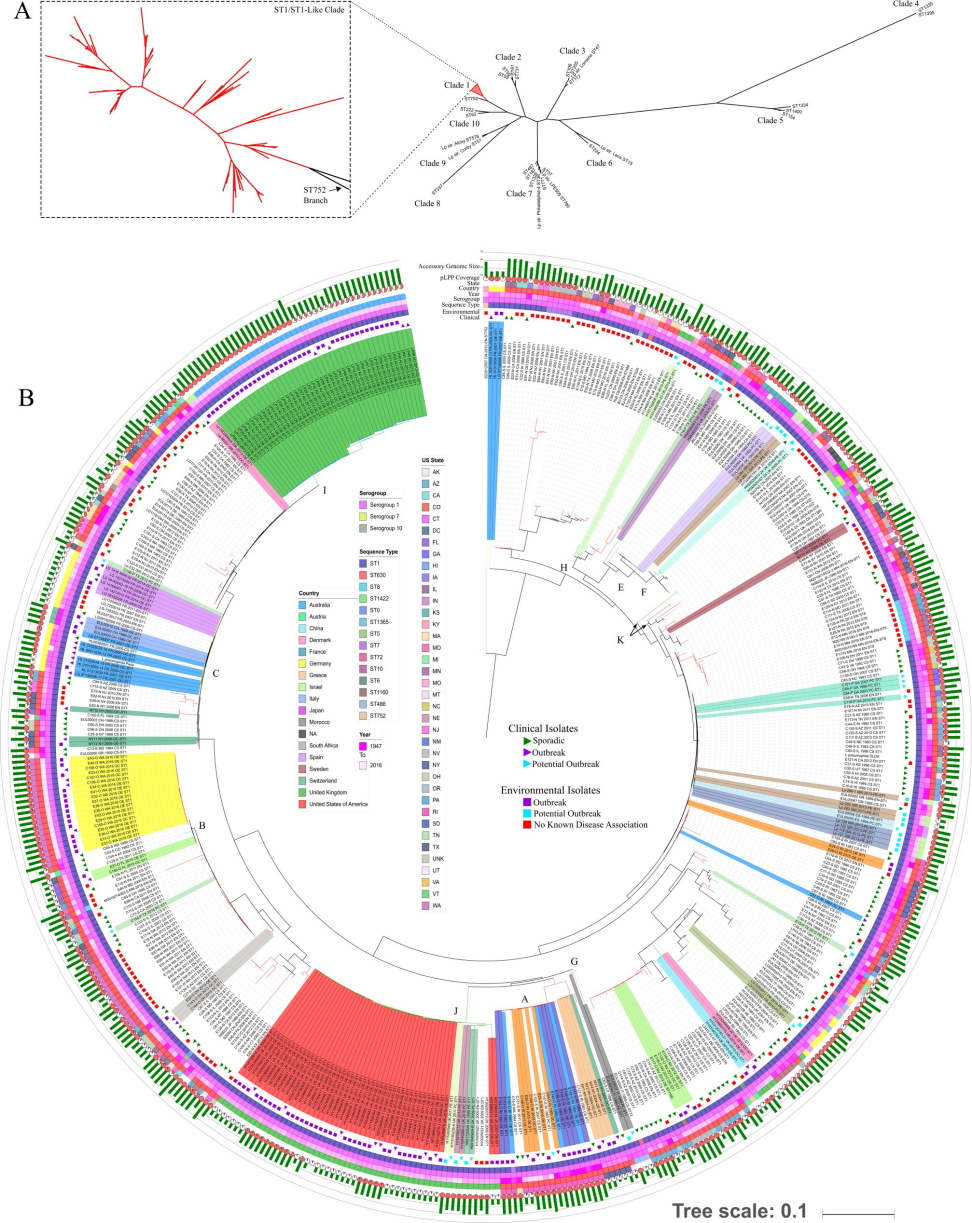
Whole genome SNP-based phylogenetic comparisons of ST1 and ST1-like isolates in the current dataset. **A**) Unrooted tree of 28 different *L*. *pneumophila* sequence types, including the ST1 clade (collapsed red triangle) alongside an international ST752 double locus variant of ST1. Clade numbers are discussed in the text. Isolate ID’s for each ST are defined in the ‘Comments’ column of S1 Table. **B**) All-SNP-based phylogenetic tree of 502 ST1 and ST-like isolates rooted on the ST752 international sequence, ‘SZ2012007’. Isolate names highlighted by outbreak or potential outbreak. Clade letter designations are discussed in the text. Additional metadata displayed on the outer rings labeled according to the included legends.

A phylogeny constructed with all identified SNPs from the current ST1 and ST1-like isolates, along with 28 additional, diverse sequence types (Fig 4A), displayed ten distinct clades, including an ST1-specific clade (clade 1; Fig 4A, inset). This condensed, ST1-specific branch contains 10 or more tightly clustered, but distinct subclades. A circular, rooted SNP-based tree of all current isolate sequences revealed additional phylogenetic structure (Fig 4B), including multiple ST1 subclades within each major branch. As expected, most strains associated with potential or confirmed LD outbreaks exhibited clustering; however, we occasionally observed outbreak-associated isolates outside of their respective outbreak clades such as the New York clinical isolate ‘NY9’, and to a lesser extent, ‘NY10’ (both outbreak ‘O4’; S1 Table), as reported previously [47]. Also noted were isolates without known epidemiological links clustered within or around putative outbreak clades, including sporadic clinical isolates from Rhode Island (‘C127-S’, ‘C147-S’, ‘C102-S’, and ‘C131-S’) and Massachusetts (‘C15-S’) within the Rhode Island multi-outbreak ‘clade A’, among others.

It should be noted that the placement of epidemiologically-linked isolates and interpretation of genetic relationships did not differ between trees constructed with all available SNPs or core SNPs; however, inclusion of all SNPs appeared to introduce additional genetic variability into the tree. We observed that isolates clustering in the same clade generally shared a similar accessory genome size and gene content (Fig 4B, outer rings) relative to the Lp strain Paris plasmid (pLPP). Yet, ST1 subgroups did not exhibit dramatically different average sequence coverage for the strain Paris plasmid (pLPP) (average pLPP coverage per genome in EN = 67%, and CS = 70%). Comparative analyses of genome sizes, plasmid conservation patterns, as well as notable phylogenetic clustering within and outside outbreak clades are further described in the Supporting Results (S1 Results).

### US state and regional ST1 populations are genetically diverse

Pockets of genetic clustering among geographically related isolates without confirmed epidemiological association have been recently described [48] and were also noted here in clades from the US Southeast (‘C45-S’, ‘C101-P’, ‘C65-P’, ‘C84-P’, ‘E68-N’, and ‘C116-P’), South Dakota (‘C64-S’, ‘C23-S’, ‘C40-S’, ‘C17-S’, ‘C19-S’, and ‘C79-S’), and Sweden (‘EUL00108’, ‘LP21’, ‘EUL00109’, ‘LP23’, and ‘EUL00104’). Therefore, we investigated genetic differentiation among geographic populations through an analysis of molecular variance (AMOVA) [49]. Overall, 4.27% of the US ST1 population genetic structure (p = 0.00248 SE±0.00050) could be explained by regional geographic categorization (S7 Table). Seventeen out of forty-five pairwise regional population comparisons exhibited significant genetic differentiation (fixation index [F_ST_] p<0.05), and every one included a region in the western US (i.e., Northwest, West, West North Central, or Southwest).

We next attempted to minimize the influence of recombination, which plays a prominent role in *L*. *pneumophila* genetic ecology [32, 46], in the AMOVA by removing all putative horizontally acquired regions and utilizing only 799 vertically inherited core SNPs. In this case, a larger proportion of the US ST1 genetic structure (~8.97%; p = 0.000 SE±0.0000) was attributed to geographic categorization. When isolates were classified by state of origin, 8.61% of nucleotide variation was attributed to geography, but removing potential recombinant SNPs had a lesser effect (11.1%). Despite US regional and state-specific genetic contributions, ST1 population structure was due in largest part (≥90%) to within group nucleotide variation (among isolate populations within regions or states).

**Fig 5:**
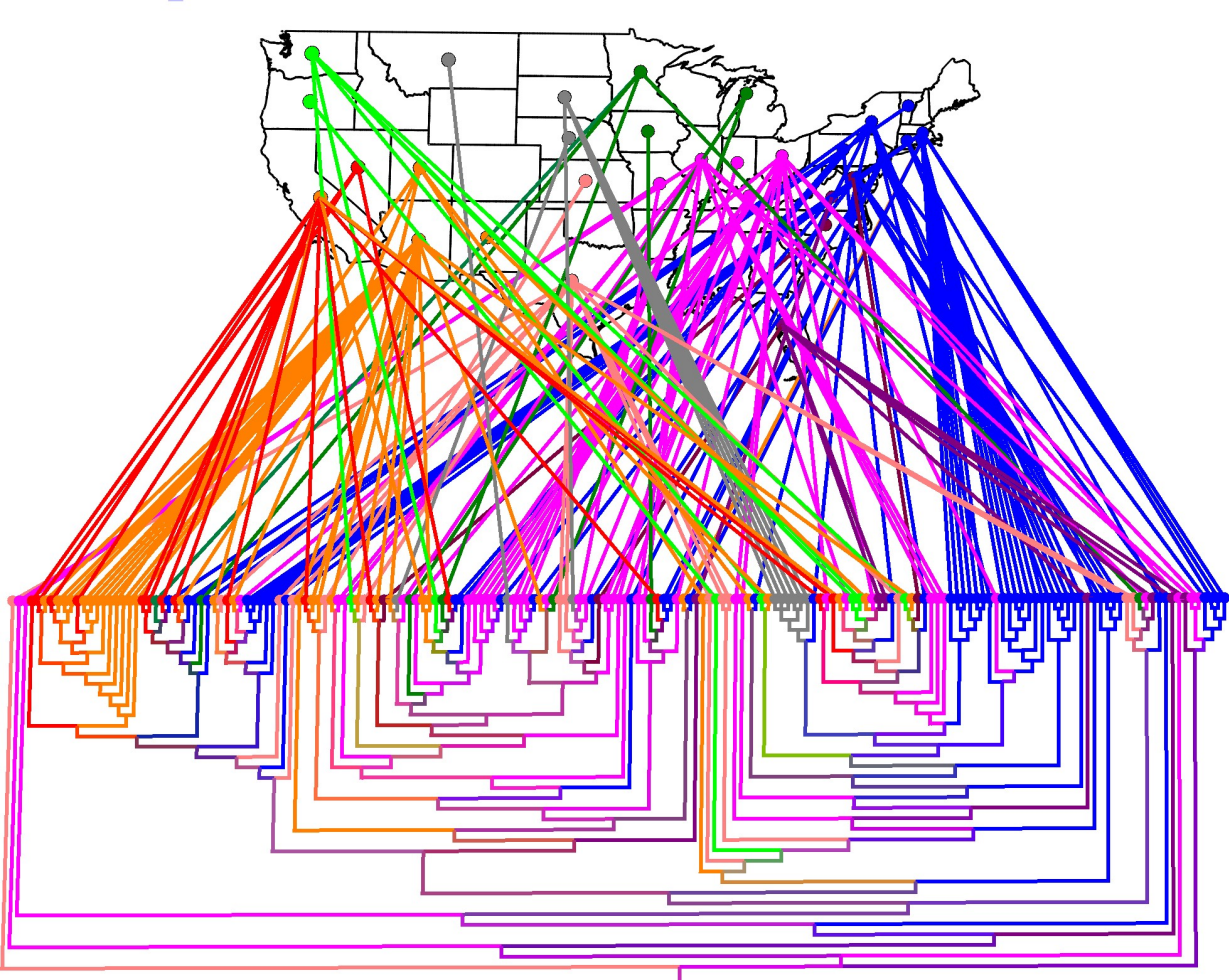
Phylo-geographical clustering of *L*. *pneumophila* serogroup 1, ST1 isolates from the US. Analysis conducted with 799 core, non-recombinant SNPs obtained from 187 isolate sequences after applying the Gubbins algorithm (See Methods). Colors were generated automatically in GenGIS to distinguish isolates based on the 9 NOAA climate regions. Regions and matching colors lines and branches include: Northwest, bright green; West North Central, grey; East North Central, dark green; Northeast, blue; West, red; Southwest, orange; South, salmon; Southeast, maroon; Central, bright pink. Isolates from Hawaii and Alaska are not represented in this figure.

Geographic, spatial mapping of isolates from an ST1 core SNP tree (Fig 5), with or without recombinant regions, revealed location-based clustering trends initially detected by AMOVA. However, geographically related isolates were not strictly concentrated in single, large clades. Instead, major clades were composed of multiple, smaller, geographically-isolated, but genetically homogeneous branches. Geographic comingling, while present throughout the entire hierarchy, was more pronounced at higher levels of the tree. For example, isolates in all three major clades mapped to the Northeast, Southeast, West, and East North Central regions, while smaller internal branches, in some instances, mapped almost entirely to the Southwest or Northeast.

## Discussion

ST1 is a worldwide-distributed sequence type and likely represents the largest and most successful *L*. *pneumophila* monophyletic group. ST1 legionellae are not prevalent among large LD outbreaks in the US, unlike ST36 and ST222 [3], but they are responsible for a majority of sporadic disease cases reported to the CDC where an ST was determined. As illustrated in recent reports [26, 28, 46], the ST1 population is subject to higher levels of recombination and background mutation compared to several prominent disease-causing STs, indicating that ST1 may not be as homogeneous as initially thought. The present study was undertaken to characterize the ST1 population at the genomic level, including an exploration of potential genetic differences between clinical and environmental isolate subgroups.

Examination of 3 ST1 subgroups (CS, EN, and OBP) revealed core genome sizes within the expected range for *L*. *pneumophila* [17, 26, 28, 50–54]. The pangenomes of 2 subgroups (CS and EN) were larger than previously reported for ST1 [28], which could be explained by the smaller dataset previously used (n = 71 versus n = 280 in the current study). While the EN pangenome was outsized compared to the CS and OBP subgroups, the EN core genome was comparatively smaller (Fig 2B), which was unexpected given the smaller EN subgroup. Additionally, a previous report suggested the accumulation of new genetic content was leveling off in the ST1 population [28], however, we find this dependent on the subgroup examined. Of central importance, the EN subgroup exhibited higher levels of genetic variation across the genome and greater accessory gene content compared to the CS and OBP subgroups. Among the potential basis for these observations are an enrichment, in either the environmental ST1 pan or accessory genomes, for components of type IV conjugative DNA transfer, transposition, and recombination. Previous studies have concluded that HGT is among the most powerful drivers of nucleotide diversification among many Lp sequence types, including ST1 [28, 32, 46, 55]. Our results support this premise and further suggest that environmental ST1 isolates are subject to more recombination events and at higher frequencies compared to the sporadic disease-associated ST1 subgroup. Plasmids represent a mobile, readily available source of accessory content that could, in part, explain genetic differences between subgroups [47], yet average coverage for the Lp strain Paris plasmid was not dissimilar between the EN and CS subgroups, and a search for alternative plasmids was not performed.

Gene ontology enrichment analysis (GO) of the shared accessory genome also suggested that all 3 ST1 subgroups retained the capacity for horizontally acquiring, and potentially donating, additional gene content. Notably, variable nucleotide regions 5–8 and 26–28 of the full ST1 alignment were contained within large genomic islands required for efficient growth in amoebal hosts and, according to a previous study, could suggest a modular genomic architecture that allows for expansion of the Lp host range [56].

The simplest, biologically-relevant hypothesis explaining quantitative differences in nucleotide variation between the EN and CS subgroups is that environmental legionellae, which are unassociated with identified human disease, inhabit diverse ecological habitats and harbor a genetic repertoire that reflects multiple, niche-specific adaptations. In contrast, legionellae recovered from clinical settings have in common a confirmed ability to cause human disease. Thus, clinical-associated isolates may represent an environmental subpopulation with increased potential for human pathogenicity arising from unknown pressures, possibly associated with habitat. Such selection could introduce molecular constraints on key virulence determinants, thus curbing gene diversification or influencing gene presence or frequency [40]. In one possible illustration of this phenomenon, half of the top 10 most enriched genes in the CS subgroup (Table 1) appear to be acetyltransferases. Thus, it is tempting to ask if this enzymatic capacity is related to *Legionella* pathogenicity through bacterial or host factor modification (e.g., LPS O-acetylation [57], and Dot/Icm-secreted effector acetyltransferases [52] [see [58] for a review]).

Several reports have explored the potential virulence-associated underpinnings of Lp isolates by classifying genetic markers that distinguish clinically significant from benign environmental legionellae [59–62]. We identified a handful of enriched loci or gene categories that may promote virulence in ST1 clinical isolates; however, our results suggest the clinical ST1 subgroup is defined largely by increased genetic conservation or homogeny compared to the environmental subgroup, and by lower comparative accessory genome enrichment for elements of DNA transfer and recombination. We did not look outside the ST1 population, therefore, our analysis does not preclude the existence of genetic loci that define clinical-associated sequence types or subpopulations outside ST1.

This study highlights the complexities of interpreting phylogenetic relationships within a common genotype, especially in the context of outbreak events. The ubiquity and environmental distribution of ST1 increases the probability that epidemiologically linked but genetically distinct ST1 isolates, coexisting with the outbreak strain, could be recovered during environmental sampling as part of an investigation (e.g., ‘E24-O’ and ‘E26-O’ were originally considered part of ‘O1’). Alternatively, the genetic homogeneity implied by a common sequence type means that isolates not epidemiologically associated with an outbreak may occasionally share the outbreak genotype (e.g., ‘C24-S’ appears related to both isolates in ‘O27’), as we recently reported for the 1976 Philadelphia outbreak [63]. Lastly, isolates genetically and geographically related to, but temporally offset from an outbreak cluster, may represent unidentified disease cases resulting from long-term *Legionella* persistence (e.g., ‘D4846’ may be an early case from the WA ‘O8’ outbreak lineage) [see [64] for review].

We also uncovered individual outbreak-associated ST1 *clinical* isolates placed outside their presumed outbreak clades (e.g., ‘C51-O’ and ‘NY9’), and *matching* clinical/environmental outbreak associated strains that clustered away from their originally assigned outbreak (e.g., ‘HL_00514008–13’, ‘HL_01313038’, and ‘LG_07135008’). This implies multiple, discrete ST1 genotypes may coexist in the same location, or may illustrate sporadic disease cases with epidemiological exposures common to an outbreak cluster. Nevertheless, it is clear from the current analysis and recent reports [28, 32, 47] that prominent regional Lp genotypes do exist, thus, genetic interpretations should rely heavily on confirmed epidemiologic associations, as illustrated by a recent LD investigation in Germany [65]. This phenomenon is not limited to ST1; different sequence types have been recovered from clinical cases in each of at least 4 LD outbreak investigations conducted by the CDC since 1982. More comprehensive, wide ranging environmental sampling could place potential outbreak associated isolates in a more accurate ecological context.

Molecular variance analysis of diverse US ST1 isolate sequences indicated that up to ~10% of population genetic structure can be attributed to geographic categorization; however, ST1 population structure is dominated by nucleotide diversity within these geographies. Recombination contributes to gene flow among ST1 strains in different states and climate regions, but nucleotide conservation, characteristic of regions or states lies, at least in part, outside these genomic loci. This small percentage of geographically-explained genetic structure was reflected in occasional phylogenetic clustering by traditional tree-based methods (Fig 4B) and phylo-geographical mapping (Fig 5). The remaining, substantial nucleotide variation not explained by geographic categories may be due to a diverse but extremely stable endemic ST1 population. Equally likely is an existing, widespread environmental mechanism for continual mixing of physically distant and dissimilar ST1 genotypes, such as ground [66, 67] and surface water transport [68], or the natural processes of atmospheric aerosolization, dispersion, and deposition [69, 70].

The isolates examined in this study comprise the largest single-ST *Legionella* sequence dataset analyzed to date, and thus provide enhanced genomic context for ST1 comparisons. However, our results should be interpreted with several potential limitations. A majority of CDC *Legionella* isolates were recovered after the year 2000. It is not clear if this uneven temporal distribution negatively impacted the present ST1 comparisons, but previous reports suggest the effects are likely minimal over this time frame given the low *Legionella* background mutation rate [28, 32]. The current ST1 isolate dataset is also not geographically complete, and US states with higher LD rates may be overrepresented. We did not examine geographic diversity among internationally-derived isolate sequences, therefore caution is recommended when extending these results to other continents or climate regions. Additionally, the classification of non-outbreak environmental isolates, which originated from routine cooling tower or potable water samples and not from natural freshwater, could introduce an unidentified genetic bias. And while no direct epidemiological link was documented among the sporadic clinical disease-associated isolates, we cannot rule out the possibility that some are associated with unidentified clusters. Finally, the recombination events identified here, while consistent with prior publications, are nonetheless predictions based on bioinformatic analysis of SNP density, and not experimentally confirmed.

Within the past 10 years, several whole genome-focused publications have included ST1 datasets [25–28, 46], however, the current study is the first to characterize genetic diversity and population structure of a large, US ST1 and ST1-like strain collection, alongside international sequences. The 289 new *L*. *pneumophila* genomes reported here contribute to a growing, more ecologically comprehensive dataset for the development of improved, rapid molecular typing methods, and for analysis of variation within *L*. *pneumophila* populations. This collection can also provide essential genetic context to support future Legionnaires’ disease outbreak investigations involving ST1 strains.

## Materials and methods

### *Legionella* culture and sequencing

To genetically characterize the US ST1 population structure, we sequenced 289 clinical and environmental Lp strains that originated from 36 US states and one international location (South Africa) and were archived at the CDC between 1977 and 2016 (S1 Table). Included were 55 isolates representing 9 confirmed or potential LD outbreaks, as well as 21 non-ST1-like isolates (i.e., not ST1 or ST1 variant). All environmental isolates were recovered from man-made cooling or potable water distribution networks and were not associated with known cases of disease. An additional 234 existing ST1, SLV (Single Locus Variant), and DLV (Double Locus Variant) isolate sequences were included from New York State, Minnesota, and from publically-available *L*. *pneumophila* isolate datasets representing geographically diverse international locations including Europe, Australia, China, Japan, and Israel [17, 19, 26, 27, 54]. The complete 502 isolate ST1 and ST1-like dataset (without the 21 non-ST1-like isolates) included 3 serogroups (sg1, 7, and 10), 15 different ST1-like SLVs and DLVs, including ST5, 6, 7, 8, 10, 72, 486, 630, 752, 1160, 1365, 1422, and 2 novel ST1-like isolates yet to be assigned an ST. The sequence collection was further divided into the following subgroups containing only non-identical serogroup 1, ST1 isolate sequences whose members are defined in S1 Table: ‘CS’—sporadic clinical disease not associated with an outbreak, (n = 139); ‘EN’—non-disease-associated environmental’ (n = 99); and either ‘OB’–confirmed outbreak-associated (n = 16); or ‘OBP’–outbreak and potential outbreak-associated (n = 28) subgroups. When available, only a single isolate sequence was included per patient or per environmental location in subgroups and during analyses. Outbreak subgroups contain only a single clinical or environmental representative of each defined outbreak.

All sequenced isolates were plated from frozen stocks and grown as previously described [63] on solid BCYE agar plates containing L-cysteine. No samples were collected for the sake of this study and all samples were anonymized prior to access.

### SBT-Based, ST1 population description and clonal complex analysis

Sequence type data were compiled for ST1 and ST1-like isolates from the European Study Group for *Legionella* Infections (ESGLI) SBT database (http://www.hpa-bioinformatics.org.uk/legionella/legionella_sbt/php/sbt_homepage.php) and from an internal CDC SBT database as of December, 2017 for comparative analyses and for examination of clonal complexes, ST1 SLVs, and DLVs through the eBURST V3 software program and visualization [71]. *Legionella* sequence type diversity within each predicted clonal complex was calculated using Simpson’s Diversity (1-‘D’) index [72]. Isolates originating from the US were removed from the ESGLI database before analysis.

### Genomic DNA extraction, NGS library preparation and sequencing

Genomic DNA (gDNA) was extracted from pure *Legionella* culture isolates as previously described [63] using the Epicenter Masterpure DNA purification Kit (cat. no. MCD85201, Epicentre, Madison, WI), as per the manufacturer’s instructions. Illumina compatible sequencing libraries were constructed with the NEBNext Ultra II DNA Library Preparation Kit (cat. no. E7370, New England Biolabs, Ipswich, MA) and MiSeq 2 x 250bp sequencing runs were performed with Illumina version 2 chemistry as previously detailed [63].

### Reference-Based sequence mapping

Paired Illumina sequencing reads were mapped against the ST1 *L*. *pneumophila* str. Paris reference genome using bowtie v.2.2.9 [73] with the settings “—very-sensitive-local”, “—no-unal”, and “-a”. Nucleotide variants were called with Freebayes v.0.9.21 [74] using the settings “-q 20”, “-p 1”, “—min-coverage 25”, “-F 0.75”, and “-j”. Indels were removed with VCFtools v.0.1.14 [75] and SNPs were recoded with VCFfilter from the vcflib package with QUAL > 1. Identified SNPs were re-mapped to the strain Paris reference using VCFtools to produce a full length reconstruction of the isolate’s chromosome in the same orientation and order as the reference genome. As SNPs were only called on sites with > = 25x coverage, we used a custom perl script to mask with “N”s all sites in the individual isolate chromosome which had lower coverage than our SNP discovery threshold. After masking low coverage regions, the average sequence coverage for any isolate relative to the Paris reference was 97.3% (SD±0.0331, Median 97.9%). Sequence mapped chromosome reconstructions were concatenated into a single FASTA file to produce a reference mapped alignment of all 501 isolates. Smaller isolate subsets, such as the non-redundant ST1 data set were constructed by subtraction from this master alignment.

### Genome assembly, gene prediction, and pangenome analyses

Illumina sequencing reads were assembled into draft contiguous sequences (contigs) using Velvet v.1.2.10 as previously described [47]. Prokka v.1.8 [76] was used with default parameters to predict and annotate rRNA, tRNA, tmRNA, and amino acid coding sequences for each newly sequenced isolate as well as for Illumina whole genome shotgun sequencing data previously published and obtained from public repositories. Gene clustering and pangenome analyses were conducted with Roary v.3.5.9 [77] along with the ‘roary_plots’ python script. Six total pangenome distribution categories were defined with an additional ‘core-1’ category that includes genes found in all isolates of a subgroup minus 1 to account for potential errors in sequencing and/or assembly. The accessory genome included all genes not found in the ‘core’ and ‘core-1’ categories. After gene prediction, annotation, and orthologous protein clustering, pan-genomes were compared, including core and accessory content, within and across subgroups. The “Core” pangenome contains all genes common to every isolate of all subgroups while the “Accessory” pangenome contains all genes found in at least one isolate of each subgroup.

### Gene Ontology (GO) and gene level enrichment analyses

Gene enrichment and gene ontology (GO) [78, 79] categorization were accomplished with two sets of gene annotations created through Interproscan v.5.24–63.0 [80]. One set was created from the *de novo* gene set predicted by Roary [77] for use in gene presence/absence comparisons. The other set was created based on the genes present in the *L*. *pneumophila* str. Paris reference genome to be used with data sets created from read mapping approaches, such as the nucleotide variability comparison sets. Gene subsets derived from pangenome and accessory genome comparisons were analyzed with Ontologizer v.2.1 [81] using an appropriate background gene set (the combined ST1 pangenome) and Benjamini-Hochberg correction (BH) [82] to identify overrepresented GO terms. Gene level enrichment analysis was carried out by combining gene presence and absence frequencies for the EN and CS subgroups to create an average frequency for each gene, against which the individual EN and CS subgroup gene frequencies were compared, initially by a 1-tailed Fisher’s exact test and then by a 2-tailed Fisher’s exact test with BH multiple testing correction.

### Phylogenetic analysis and visualizations

Phylogenetic trees were constructed and visualized as previously described [63] by the parsimony method using kSNP v.3 [83] and the Interactive Tree of Live (iTOL; http://itol.embl.de/) [84]. Additional figure labels and detail were added with InkScape v.0.48.5 (https://inkscape.org/en/).

### Characterization of nucleotide diversity and potential recombination events

A custom sliding window approach (window size = 500 nt, step size = 250 nt) was used to assess nucleotide variation along the length of the reference-mapped alignment. For every pairwise comparison of isolates, we summed the number of SNP differences and tracked the number of conserved sites within the window. If a gap or unresolved character was present at a site in either or both isolates, that site was counted neither as a mismatch nor a conserved site for that pair. The nucleotide variability value for the window was calculated as follows:

$$nucleotide\;variability=\frac{\sum \;mismatches\;in\;every\;pairwise\;comparison\;at\;a\;single\;nt\;position}{number\;of\;sites\;compared\;(mismatch+conserved)}$$

To assess whether clinical sporadic or environmental isolates were accumulating variation in different genomic regions, we examined intra-group variation by conducting pairwise analyses between isolates within the same *a priori* defined group (environmental isolates vs clinical sporadic isolates) producing a group-specific variability number. Thus, the intra-group variation for environmental isolates is derived from all possible comparisons of environmental isolates, and the intra-group variation of clinical samples is derived from all possible comparisons of clinical isolates, but no comparisons of clinical to environmental isolates are included in either metric. We also investigated whether clinical sporadic and environmental isolates encompassed genomic regions that segregated into a set of related clinical-specific alleles and/or environmental-specific alleles. Inter-group analyses were conducted in a similar manner as within-groups, except that only clinical-environmental isolate pairwise comparisons were included. All possible combinations of environmental and clinical pairs were included in this value; however, no clinical-clinical or environmental-environmental pairs were analyzed. This metric is most useful when compared against intra-group variability or overall variability metrics. For example, low values of intra-group variability appearing within the same window as high between group variability suggests that there is segregation of alleles between clinical and environmental samples and that these alleles are notably different from each other.

High nucleotide variability was identified by filtering and merging the top 3% of variable windows within 5000 bases of each other to produce contiguous regions. The predicted variable region size and distance cutoffs were selected to maximize region nucleotide length but minimize the number of variable regions and the fraction of the total genome incorporated. Measurements of nucleotide diversity (d) within subgroups was accomplished with MegaCC v.7.00 (http://www.megasoftware.net/) [85] using the *L*. *pneumophila* str. Paris reference based multiple sequence alignment of all isolates in a single subgroup. Potential recombination events were identified with Gubbins v.1.4.1 [86] as previously described [63] using the *L*. *pneumophila* str. Paris reference based alignment of all isolate sequences as input. For interpreting recombination predictions, ST1 mapping coverage was calculated at each of the 14,013 overlapping nucleotide windows previously described.

### AMOVA and phylo-geographical clustering and analysis

Analysis of molecular variance (AMOVA) was conducted with the Arlequin v.3.5 software package [49, 87] using the haplotypic format after categorizing isolate sequences by US state or NOAA climate region (https://www.ncdc.noaa.gov/monitoring-references/maps/us-climate-regions.php). AMOVA was run with default parameters except that ≥ 1000 permutations were performed. AMOVA was carried out using 20,718 core SNPs from non-clonal, sg1, US ST1 isolates (n = 187 isolate sequences). The ‘Alaska’ geographic category was not considered in the final interpretation because it contained only a single isolate sequence. Phylogeographical clustering was performed and visualized with GenGIS v.2.4.1 [88] by overlaying a US ST1 phylogenetic tree with potential recombination events removed (using Gubbins) plotted to the midpoint coordinate for each US state.

### *L*. *pneumophila* reference genomes

Additional reference quality genomes used in the current study were obtained from NCBI and include *L*. *pneumophila* str. OLDA (CP016030.2), *L*. *pneumophila* str. Paris (NC_006368.1), *L*. *pneumophila* str. Alcoy (NC_014125), *L*. *pneumophila* str. Corby (NC_009494), *L*. *pneumophila* str. Philadelphia-4 (NZ_CP015931), *L*. *pneumophila* str. LPE509 (NC_020521), *L*. *pneumophila* str. Lens (NC_006369), and *L*. *pneumophila* str. Lorraine (NC_018139).

### Data access

Sequencing data derived from this study have been deposited with links to BioProject accession number PRJNA423272 in the NCBI BioProject Database (https://www.ncbi.nlm.nih.gov/bioproject/). Raw Illumina sequencing reads were assigned the SRA accession SRP127407 (Sequence Read Archive, https://www.ncbi.nlm.nih.gov/sra) and individual isolate SRA sequence accession IDs are listed in S1 Table.

## Supporting information

S1 Results Supplemental results and findings. (DOCX)

S1 Table List of *L. pneumophila* strains or isolate sequences utilized in the present study. (XLSX)

S2 Table Enriched GO annotation terms. (XLSX)

S3 Table Venn overlapping subgroup genes. (XLSX)

S4 Table ST1 variable nucleotide regions (3%, 5000bp). (XLSX)

S5 Table EN/CS absolute comparison regions meeting variability threshold (3%, 5000bp). (XLSX)

S6 Table GO term enrichment with combined and individual EN Subgroup variable regions. (XLSX)

S7 Table AMOVA analysis. (XLSX)

S1 Fig ST1 and ST1-like clonal complex and locus variants. **A**) ST1-founded clonal complex reconstruction by eBURST using data from the combined CDC and ESGLI SBT databases, as of December, 2017. Non-ST1 sequence types found in the CDC SBT database are highlighted in blue and STs shared between the ESGLI and CDC collections are highlighted in red. **B**) ST1 and ST1-like single and double locus variant entries in both the CDC and ESGLI databases as a fraction of the total number of SBT entries as of December, 2017.
(PDF)

S2 Fig *L. pneumophila* ST1 and ST1-like core and accessory gene composition and comparative GO term enrichment among subgroups meeting the threshold for statistical significance (p<0.05). (PDF)
