# Supplementary material for: Genomic heterogeneity differentiates clinical and environmental subgroups of Legionella pneumophila sequence type 1
Source: PLoS One. 2018 Oct 18;13(10):e0206110. doi: 10.1371/journal.pone.0206110 (PMC6193728; doi:10.1371/journal.pone.0206110)
Supplement: S1 Results — (DOCX) [file pone.0206110.s001.docx]

Genomic heterogeneity differentiates clinical and environmental subgroups of *Legionella pneumophila* sequence type 1

Jeffrey W. Mercante, Jason A. Caravas, Maliha K. Ishaq, Natalia A. Kozak-Muiznieks, Brian H. Raphael, and Jonas M. Winchell

Pneumonia Response and Surveillance Laboratory, Respiratory Diseases Branch, Centers for Disease Control and Prevention, Atlanta, GA, USA

# **S1 Results**

## ST1 Clonal Complex and Locus Variant Analyses

A large, ST1-founded clonal complex was observed in both the EWGLI and CDC isolate databases (data not shown), which contained the largest single collection of isolates (ESGLI/EWGLI, n=5,395, 49%; CDC, n=391, 38%) among all predicted clonal groups. These major ST1 complexes encompassed 51% and 9% of the total non-outbreak associated STs deposited in the ESGLI/EWGLI and CDC databases, respectively. The combined ST1 clonal complex population (ESGLI/EWGLI + CDC collections) contained 6069 isolates and was marginally more heterogeneous (Simpsons Diversity [1-D’] = 0.988, see Materials and Methods) than the clonal complexes founded by ST23 (1-D’=0.805), ST154 (1-D’ = 0.914), andST42 (1-D’ = 0.703); these findings are consistent with a recent study suggesting that the ST1 group may be relatively heterogeneous [1] compared to other highly represented, clinically-relevant ST’s in Europe. Notably, there are a disproportionately large number of ST1 single locus variants (SLV; n=63; S1B Fig) compared to other large clonal complexes (e.g., ST154, n=14 SLVs; ST23, n=39 SLVs; and ST42, n=18 SLVs) in the combined collection. Single and double locus variants of ST1 make up ~4% of all strains assigned an ST from the combined EWGLI and CDC collections, and, combined with ST1, they make up 18.2% of all non-outbreak associated isolates. Of the 1,251 STs in the combined ST1 clonal complex, only 54 are shared between the ESGLI/EWGLI and CDC databases, and 51 are exclusive to the CDC collection (red and blue labels in S1 Fig).

## Additional ST1 Subgroup GO and Gene Level Enrichment Analyses

Direct comparison of all predicted pangenomes (Fig 3B) revealed a shared core of 2702 genes (S3 Table) significantly enriched for 59 GO terms encompassing 1406 unique genes. The CS and OBP pangenomes were enriched for 34 and 44 GO terms compared to the combined ST1 pangenome, encompassing 1672 and 1580 unique genes, respectively. In contrast, the EN pangenome was enriched for only a single term, ‘oxidoreductase activity’, that included 248 unique genes involved in diverse bacterial functions such as respiration (e.g., *ctaD*), detoxification (e.g., *sodB*), and lipid metabolism (e.g., *fadJ*), among others.

The CS and OBP subgroups shared 67 core genes also found in the EN accessory genome, but only a single EN core gene, *rmlC*, was included in the CS and OBP accessory genomes; no significant GO enrichment was documented in either comparison. While not experimentally confirmed in *Legionella*, *rmlC* is reported to be involved in LPS core oligosaccharide biosynthesis as part of the dTDP-rhamnose pathway [2], and is exclusively expressed in the exponential phase of growth [3]. Genes shared among the OBP core and EN and CS accessories (85 genes) were enriched for 38 GO terms and encoded gene products involved in ATP metabolism, such as synthases and ATPases (e.g., *atpA* and *atpG*), and efflux of metals such as copper, silver, zinc, and cadmium (e.g., *cusA* and *czcA*), among others. The exclusive accessory genome shared between the CS and EN subgroups (301 genes) exhibited GO enrichment categories also found in the all-subgroup shared accessory and EN-specific accessory genomes such as transposition-, and nucleic acid binding-related. The small, shared OB and EN accessory genome (12 genes) was significantly enriched for one category, ‘transposase activity’.

The larger group of 11 enriched genes (Table 1, Group 1; found in 26% of the EN genomes but only 7% of CS genomes) mentioned in the main text contained elements of a P-Type IVA conjugal DNA transfer system (e.g., *trbC* and *traG*) similar, but not identical to subregions 1/I and 2/I of the *L. pneumophila* str. Corby Trb genomic islands [4], and subsequently identified in *L. pneumophila* str. Lorraine and *L. longbeachae* str. NSW [5]. Only *L. pneumophila* str. ST23 (accession ID: NZ_LT632615) encodes a Trb island that is structurally identical to this ST1 region, which is located immediately downstream of a *csrA* paralog from position 2,712,548 – 2,731,469. The second, smaller enriched/depleted gene grouping (Table 1, Group 2) included 2 predicted genes (found in ~19% of EN genomes versus ~3% of CS genomes) that function in DNA integration and recombination (*xerC* recombinase/integrase and *hin* DNA invertase). The remaining enriched genes encoded a hypothetical protein (~13% of EN genomes) found in many non-ST1 *L. pneumophila* strains, and a transposase (found in 80% of EN genomes) whose sequence is highly conserved in approximately half of our ST1 isolates, but was only identified with complete coverage and lower identity (77%) in one additional *L. pneumophila* isolate, ST222 strain Toronto-2005 [6].

Only 2 publically available *L. pneumophila* genomes (reference strains Paris and OLDA), both ST1, displayed identical genomic architecture and sequence to the CS genomic region described in the Results, which is located from position 3,386,173 – 3,405,722 relative to strain Paris (data not shown). Encoded in this region were components of the twin-arginine translocation system (Tat) previously found to be important for intracellular replication and biofilm formation [7, 8]. The majority of the later fifth of this CS region, that encompasses most of these CS genetic features, has no sequence homology outside of existing ST1 reference sequences.

A handful of enriched genes and GO terms were identified when all VNRs were combined into a single data set (S2 Table), as well as specific GO enrichment in a single variable region (VNR18); however, most genes in these GO categories were involved in biotin metabolism and found at a single genomic locus, which likely created the appearance of significant enrichment across the genome.

## Additional Details of Variable Nucleotide Regions

In addition to pP36, multidrug transporters, and the LPS biosynthesis loci, the following genetic features were noted within VNRs: the LGI-1 genomic island-associated Type IVA secretion system and the tailing end of a larger genomic island (LppGI-1) (VNR14 and 15, respectively; Fig 3A “Conjugation Machinery”) [9], an adenylate cyclase-like protein (VNR20), the LppGI-2 genomic island (VNR29-32) [10], and the integration host factor beta subunit (*ihf*B; VNR33) that is required for growth and pathogenicity in at least one amoebal host [11]. The largest contiguous region with the highest average nucleotide variability, VNR21 (Fig 3A “VNR21”), was located immediately downstream of a flagellar biosynthesis locus and encompassed genes for DNA recombination and repair, as well as fatty acid and lipid transport.

GO term category analysis failed to uncover any significant enrichment within either the combined or individual CS variable regions. But similar to previous enrichment analysis for the full dataset, most genes in 5 of the 6 EN-specific enriched categories were concentrated in a single EN variable region (EN-VNR13), and these enriched GO categories included factors for heme/iron transport, cofactor transport, and protein biogenesis. The 6^th^ GO term, “electron carrier activity”, encompassed 8 genes that were distributed among 4 separate genomic locations; genes in this category included the electron transport-associated cytochromes b and c, and flavoproteins, as well as ferredoxin.

## Features of Selected Recombination Regions and Low Coverage Areas

Recombination region R5b/c also encompassed genes for long-chain fatty acid transport (lpp1773), and de novo pyrimidine biosynthesis (lpp1784, *pyrD*), among others. The features encoded within the R6 recombination locus (lpp2053-2058) were less defined. In addition to hypothetical proteins, one putative transposase was identified, which is more traditionally associated with mobile elements. Of note, one hypothetical protein, encoded by lpp2058, was previously predicted to contain an ankyrin repeat [5] and may have been acquired by horizontal gene transfer (HGT). Thus, it was speculated to be important for *Legionella* pathogenicity and the establishment of an intracellular niche.

As described in the main text, 4 distinct low coverage (LC) regions were found in the ST1 reference-mapped alignment. LC1, composed of 2 closely situated coverage dips, encompassed at least one Type IVA secretion system component (TraK), as well as an integrase and an RND transporter; LC2, which exhibited the lowest coverage of all regions examined (30-80%), fell entirely within the sg1-specific 18kb LPS biosynthesis region; and LC3 and 4, in part, paralleled the previously described LppGI-1 and LppGI-2/LGI-2 genomic islands, respectively. LC1-3 (and the region immediately upstream of locus 4) align with VNRs identified above, but only LC1 and 2 correspond to potential recombination hotspots.

## Additional Analyses of ST1 and ST1-Like Isolate Phylogenetic Clustering

Among the 8 non-ST1 clades that displayed relatively tight clustering were clade 2 (STs 731, 161, and 59), clade 3 (ST’s 109, 205, 47, and 117), clade 4 (ST’s 1335 and 1339), clade 5 (ST’s 1134, 1400, and 154), clade 7 (ST’s 37, 780, 771, 36, 1326, 187, and 487), clade 9 (ST’s 578 and 51), and clade 10 (ST’s 222 and 62).

Additional examples of extra-outbreak clade localization within the ST1 and ST1-like circular, rooted phylogenetic tree include 3 French ‘012’ isolates from the 2000’s (‘HL00514008-13’, ‘HL01313038’, and ‘LG07135008’); a collection of potential outbreak-associated environmental isolates from Israel (potential outbreak ‘P13’); 4 isolates from a potential cluster (‘P14’) at the same Texas facility; a single Washington State isolate associated with ‘O8’; and 3 Rhode Island isolates (‘C51-O’, ‘E24-O’ and ‘E26-O’) representing 2 ostensibly independent but geographically related outbreaks separated by 22 years.

Further examples of epidemiologically unassociated isolates clustering near outbreak clades include an older environmental isolate from Florida (‘E104-N’) that fell within a confirmed FL outbreak from 2015 (clade ‘B’); sporadic clinical (‘HL00364001’) and environmental isolates (‘LG11054025’), and *L. pneumophila* str. Paris that clustered among 2 French outbreaks (‘O12’ and ‘O13’; clade ‘C’); and a large, mainly Southwest US clade in which an entire Nevada-based outbreak (‘E118-O’, ‘C132-O’, ‘E116-O’, and ‘E117-O’) was situated. Among unconfirmed outbreaks, 5 Danish isolates (‘EUL00082’, -84, -85, -88, and -89) clustered among a potential outbreak in the same country (‘P9’) (clade ‘E’); 1 isolate from the UK (‘H102860194’) clustered with ‘P7’ (clade ‘F’); and 1 isolate from Italy (‘EUL00045’) clustered with ‘P11’ (clade ‘G’).

## Comparative Analysis of Genome Size and Plasmid Conservation Patterns

As described in the main text, phylogenetically clustered isolates (Fig 4B) shared similar accessory genome size and gene content relative to the strain Paris plasmid. For example, all 6 isolates in ‘O13’ individually encoded ~346 accessory genes and ~5% of the Paris plasmid (clade ‘C’), and 18 of 20 isolates in O8 (clade ‘B’) possessed ~482 accessory genes along with >99% of pLPP. However, this trend was not universal, and isolates within outbreak or potential outbreak clades occasionally exhibited divergent phenotypes, including isolates HL01013003-14 and HL 01313039 in the main French ‘O12’ clade (clade ‘B’), the UK ‘P6’ isolate pair (clade ‘H’), several isolates in ‘P13’ from Israel (clade ‘F’), the Italian ‘P11’ isolates (clade ‘G’), and 2 isolates from Rhode Island ‘O1’ (clade ‘A’). Distinct, branch-specific genome size and plasmid conservation patterns were evident within the larger outbreak clades. At least 3 patterns were observed in ‘O9’ from Australia (clade ‘I’) that mirrored the branch architecture of the outbreak clade, and ‘O1’4 from the UK displayed 2 major patterns repeated across several distinct branches (clade ‘J’). Strikingly, at least one major branch (that includes clades ‘J’, ‘A’, and ‘G’) was composed almost entirely of isolates associated with confirmed or potential outbreaks. The clade was composed of 1 minor and 2 major branches each with 5 presumably independent outbreaks. Both major clades were comprised exclusively of isolates from the US (mostly Northeastern region) or the UK.

## Virulence Determinants within Regions of Differential Genetic Conservation

As we previously described, the CS subgroup generally displayed lower levels of nucleotide diversity compared the EN subgroup. One additional data point indicating the potential importance of genetic conservation to clinical-associated ST1 legionellae comes from additional analysis of genomic regions where nucleotide variation is restricted to only one subgroup (Fig 3B and data not shown). Of 18 distinct genomic loci exhibiting diversity almost entirely in the EN subgroup (and thus were highly or completely conserved in the CS subgroup), 14 loci included genes for known or putative type IV secretion substrates or machinery (e.g., *lem*11, *sdhA*, and *icmF*), toxins (*rtxA*), components of LPS biosynthesis (e.g., *lpxL* and *kdsA*), or other virulence associated factors (e.g., *lidL* and *ihfB*) [12-15]. Two additional regions fell within the LppGI-2 and LGI-2 mobile genetic islands [9, 10]. Yet, only 1 of 4 genomic loci with higher CS diversity contained virulence-associated features, and this single CS region fell within the previously identified variable genomic locus 1 (VGR-1) [16]. Further analysis of gene content within regions of differential genomic variation among clinical and environmental subgroups is ongoing in our laboratory and may help clarify these observations in the future.

# **References**

1. David S, Rusniok C, Mentasti M, Gomez-Valero L, Harris SR, Lechat P, et al. Multiple major disease-associated clones of *Legionella pneumophila* have emerged recently and independently. Genome Res. 2016.

2. Jiang XM, Neal B, Santiago F, Lee SJ, Romana LK, Reeves PR. Structure and sequence of the *rfb* (O antigen) gene cluster of *Salmonella* serovar *typhimurium* (strain LT2). Mol Microbiol. 1991;5(3):695-713.

3. Aurass P, Gerlach T, Becher D, Voigt B, Karste S, Bernhardt J, et al. Life Stage-specific Proteomes of *Legionella pneumophila* Reveal a Highly Differential Abundance of Virulence-associated Dot/Icm effectors. Mol Cell Proteomics. 2016;15(1):177-200.

4. Glockner G, Albert-Weissenberger C, Weinmann E, Jacobi S, Schunder E, Steinert M, et al. Identification and characterization of a new conjugation/type IVA secretion system (*trb*/*tra*) of *Legionella pneumophila* Corby localized on two mobile genomic islands. Int J Med Microbiol. 2008;298(5-6):411-28.

5. Gomez-Valero L, Rusniok C, Jarraud S, Vacherie B, Rouy Z, Barbe V, et al. Extensive recombination events and horizontal gene transfer shaped the *Legionella pneumophila* genomes. BMC Genomics. 2011;12(1):1-24.

6. Rao C, Guyard C, Pelaz C, Wasserscheid J, Bondy-Denomy J, Dewar K, et al. Active and adaptive *Legionella* CRISPR-Cas reveals a recurrent challenge to the pathogen. Cell Microbiol. 2016;18(10):1319-38.

7. Rossier O, Cianciotto NP. The *Legionella pneumophila tatB* Gene Facilitates Secretion of Phospholipase C, Growth under Iron-Limiting Conditions, and Intracellular Infection. Infect Immun. 2005;73(4):2020-32.

8. De Buck E, Maes L, Meyen E, Van Mellaert L, Geukens N, Anne J, et al. *Legionella pneumophila* Philadelphia-1 *tatB* and *tatC* affect intracellular replication and biofilm formation. Biochem Biophys Res Commun. 2005;331(4):1413-20.

9. Wee BA, Woolfit M, Beatson SA, Petty NK. A distinct and divergent lineage of genomic island-associated Type IV Secretion Systems in *Legionella*. PLoS One. 2013;8(12):e82221.

10. Lautner M, Schunder E, Herrmann V, Heuner K. Regulation, integrase-dependent excision, and horizontal transfer of genomic islands in *Legionella pneumophila*. J Bacteriol. 2013;195(7):1583-97.

11. Morash MG, Brassinga AKC, Warthan M, Gourabathini P, Garduño RA, Goodman SD, et al. Reciprocal Expression of Integration Host Factor and HU in the Developmental Cycle and Infectivity of *Legionella pneumophila*. Appl Environ Microbiol. 2009;75(7):1826-37.

12. Gomez-Valero L, Rusniok C, Cazalet C, Buchrieser C. Comparative and Functional Genomics of *Legionella* Identified Eukaryotic Like Proteins as Key Players in Host–Pathogen Interactions. Front Microbiol. 2011;2:208.

13. Cirillo SL, Bermudez LE, El-Etr SH, Duhamel GE, Cirillo JD. *Legionella pneumophila* entry gene *rtxA* is involved in virulence. Infect Immun. 2001;69(1):508-17.

14. Lomma M, Gomez Valero L, Rusniok C, Buchrieser C. *Legionella pneumophila* - Host Interactions: Insights Gained from Comparative Genomics and Cell Biology. Genome Dyn. 2009;6:170-86.

15. Fonseca MV, Swanson MS. Nutrient salvaging and metabolism by the intracellular pathogen *Legionella pneumophila*. Front Cell Infect Microbiol. 2014;4:12.

16. Mercante JW, Morrison SS, Desai HP, Raphael BH, Winchell JM. Genomic Analysis Reveals Novel Diversity among the 1976 Philadelphia Legionnaires' Disease Outbreak Isolates and Additional ST36 Strains. PLoS One. 2016;11(9):e0164074.
