# Supplementary material for: Genomic heterogeneity differentiates clinical and environmental subgroups of Legionella pneumophila sequence type 1
Source: PLoS One. 2018 Oct 18;13(10):e0206110. doi: 10.1371/journal.pone.0206110 (PMC6193728; doi:10.1371/journal.pone.0206110)
Supplement: S1 Fig — A) ST1-founded clonal complex reconstruction by eBURST using data from the combined CDC and ESGLI SBT databases, as of December, 2017. Non-ST1 sequence types found in the CDC SBT database are highlighted in blue and STs shared between the ESGLI and CDC collections are highlighted in red. B) ST1 and ST1-like single and double locus variant entries in both the CDC and ESGLI databases as a fraction of the total number of SBT entries as of December, 2017. (PDF) [file pone.0206110.s009.pdf]

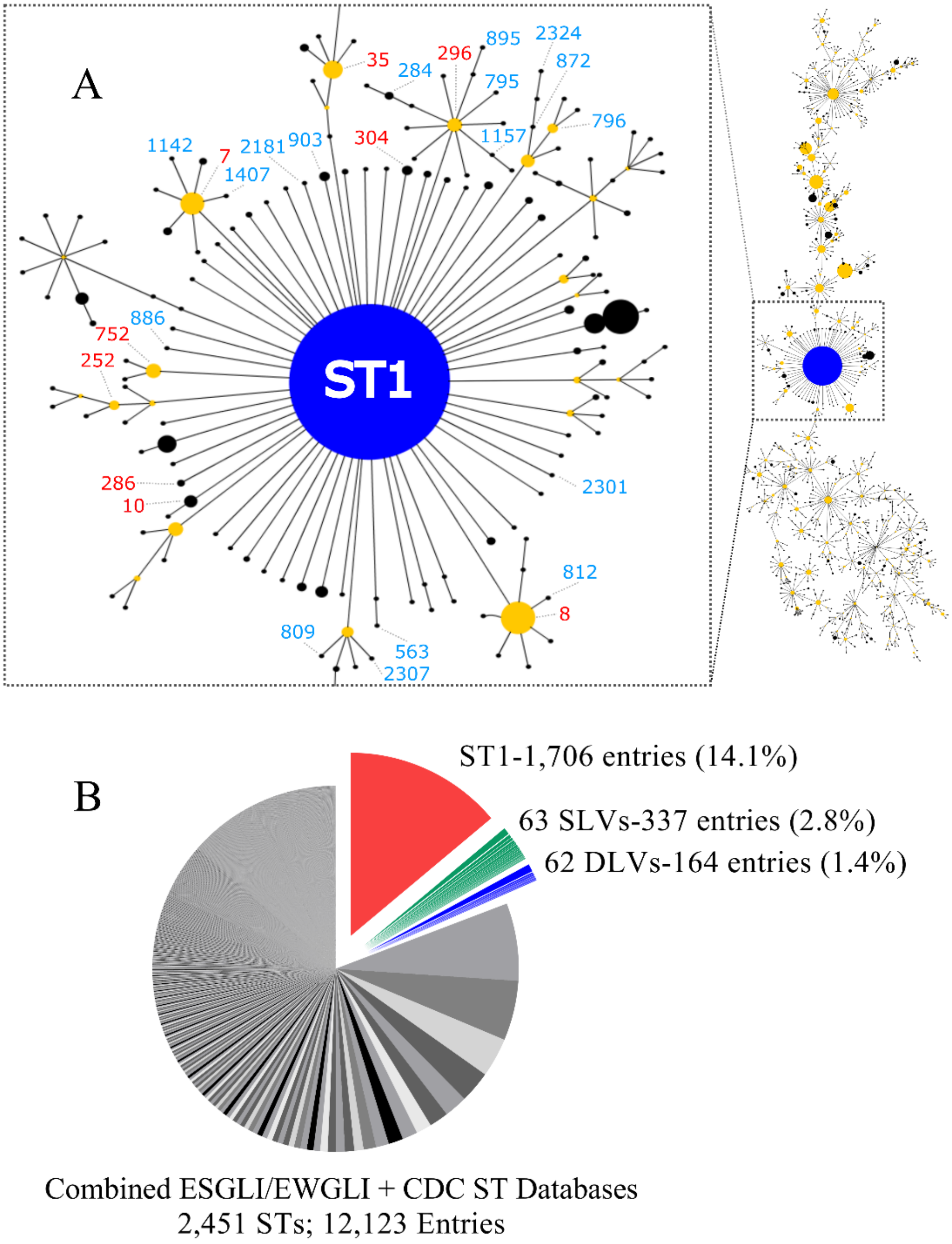

S1 Fig. ST1 and ST1-like clonal complex and locus variants. A) ST1-founded clonal complex reconstruction by eBURST using data from the combined CDC and EWGLI/ESGLI SBT databases. Non-ST1 sequence types found in the CDC SBT database are highlighted in blue and STs shared between the ESGLI/EWGLI and CDC collections are highlighted in red. **B)** ST1 and ST1-like single and double locus variant entries in both the US and EWGLI databases as a fraction of the total number of SBT entries. Data in both ‘A’ and ‘B’ is current as of December, 2017.
