# Supplementary material for: Genomic heterogeneity differentiates clinical and environmental subgroups of Legionella pneumophila sequence type 1
Source: PLoS One. 2018 Oct 18;13(10):e0206110. doi: 10.1371/journal.pone.0206110 (PMC6193728; doi:10.1371/journal.pone.0206110)
Supplement: S2 Fig — (PDF) [file pone.0206110.s010.pdf]

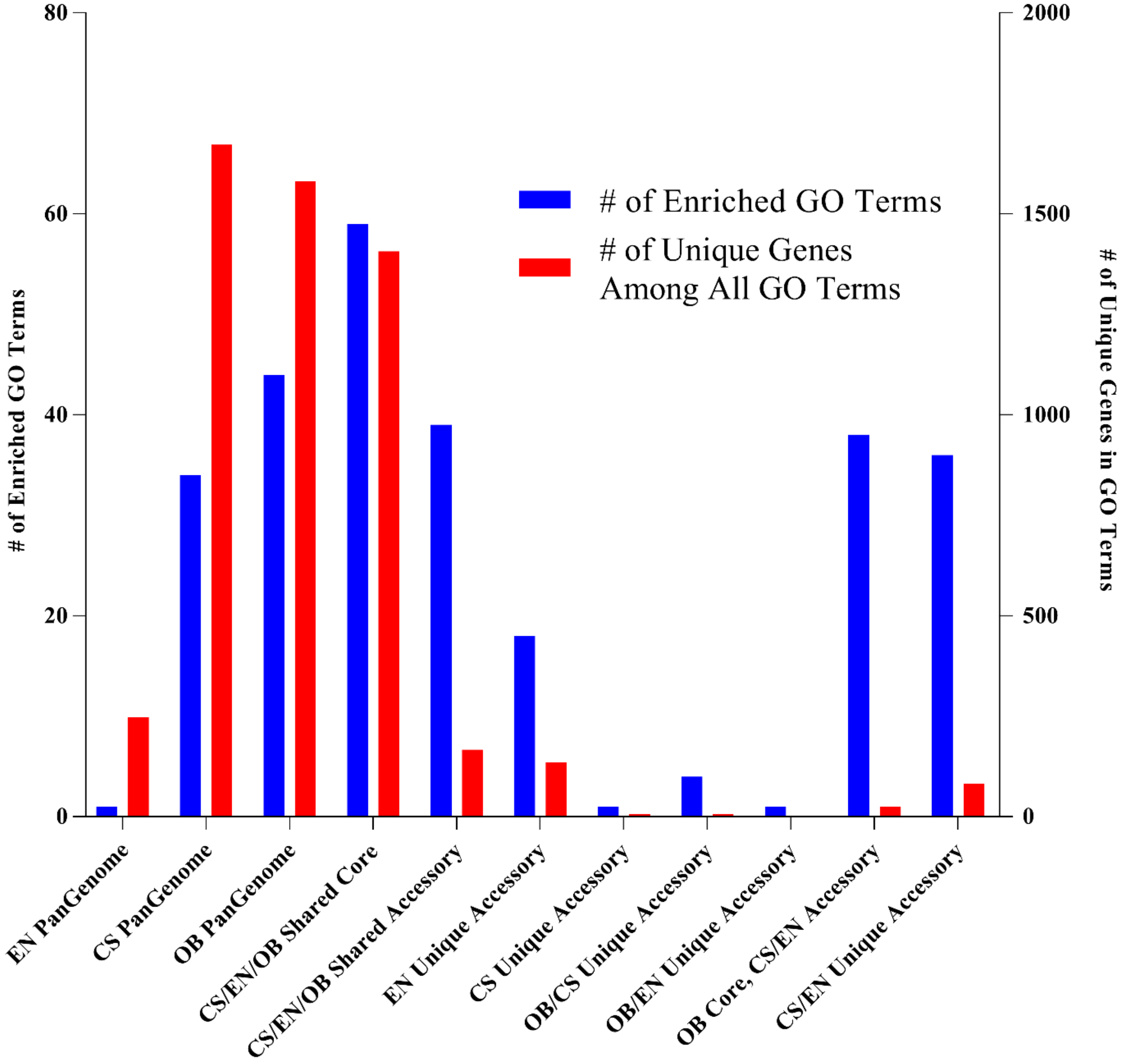

S2 Fig. *L. pneumophila* ST1 and ST1-like core and accessory gene composition and comparative GO term enrichment among subgroups meeting the threshold for statistical significance (p<0.05).
